# Bulk-like dielectric and magnetic properties of sub 100 nm thick single crystal Cr_2_O_3_ films on an epitaxial oxide electrode

**DOI:** 10.1038/s41598-020-71619-1

**Published:** 2020-09-07

**Authors:** N. M. Vu, X. Luo, S. Novakov, W. Jin, J. Nordlander, P. B. Meisenheimer, M. Trassin, L. Zhao, J. T. Heron

**Affiliations:** 1 Department of Materials Science and Engineering, University of Michigan, Ann Arbor, MI 48109 USA; 2 Department of Physics, University of Michigan, Ann Arbor, MI 48109 USA; 3 Department of Physics, Auburn University, Auburn, AL 36849 USA; 4 Department of Materials, ETH Zürich, Vladimir-Prelog-Weg 4, 8093 Zurich, Switzerland

**Keywords:** Materials science, Ferroelectrics and multiferroics, Magnetic properties and materials

## Abstract

The manipulation of antiferromagnetic order in magnetoelectric Cr_2_O_3_ using electric field has been of great interest due to its potential in low-power electronics. The substantial leakage and low dielectric breakdown observed in twinned Cr_2_O_3_ thin films, however, hinders its development in energy efficient spintronics. To compensate, large film thicknesses (250 nm or greater) have been employed at the expense of device scalability. Recently, epitaxial V_2_O_3_ thin film electrodes have been used to eliminate twin boundaries and significantly reduce the leakage of 300 nm thick single crystal films. Here we report the electrical endurance and magnetic properties of thin (less than 100 nm) single crystal Cr_2_O_3_ films on epitaxial V_2_O_3_ buffered Al_2_O_3_ (0001) single crystal substrates. The growth of Cr_2_O_3_ on isostructural V_2_O_3_ thin film electrodes helps eliminate the existence of twin domains in Cr_2_O_3_ films, therefore significantly reducing leakage current and increasing dielectric breakdown. 60 nm thick Cr_2_O_3_ films show bulk-like resistivity (~ 10^12^ Ω cm) with a breakdown voltage in the range of 150–300 MV/m. Exchange bias measurements of 30 nm thick Cr_2_O_3_ display a blocking temperature of ~ 285 K while room temperature optical second harmonic generation measurements possess the symmetry consistent with bulk magnetic order.

## Introduction

Magnetoelectric and multiferroic heterostructures have been of interest due to their potential for low-power, non-volatile spintronic devices utilizing the electric field control of magnetism^1–7^. Antiferromagnetic Cr_2_O_3_ is a promising candidate for such applications. It is one of the few single-phase materials that demonstrates an uncompensated surface magnetization that is switchable by its intrinsic magnetoelectric effect at room-temperature^3,8^. This materials configuration opens a diverse set of ways to create energy efficient spintronic devices^8–15^.

One issue hindering electric field manipulation of magnetic order in thin film Cr_2_O_3_ is the existence of twin domain boundaries that result from the growth on elemental metal electrodes, particularly in films below 250 nm^12^ which are necessary for technological adoption. The relatively conductive twin boundaries lead to high leakage current and reduce dielectric breakdown voltage down below the critical magnetoelectric switching voltage. In previous reports, this issue has been circumvented by utilizing Cr_2_O_3_ films of large thickness^12,16^ but at proposed device scales this is not a viable solution. Using a V_2_O_3_ electrode layer, a metallic oxide isostructural with Cr_2_O_3_, has been shown to reduce or even eliminate twin domains and thereby reduce the leakage current of a 300 nm thick Cr_2_O_3_ film in comparison with metal electrodes^8,17^. In this work, we investigate the DC dielectric and magnetic properties of very thin (30–60 nm) single crystalline Cr_2_O_3_ films on V_2_O_3_ thin film electrodes at room temperature. Leakage data shows robust bulk like behavior for 60 nm thick samples with electrodes below 60 µm in diameter and 6–8 orders of magnitude lower leakage current than twinned Cr_2_O_3_ films grown on (111)-oriented Pt electrodes. Finally, investigation of the magnetic properties of single crystal Cr_2_O_3_ thin films using an exchange coupled ferromagnetic layer and optical second harmonic generation indicates bulk like behavior around room temperature in films at 30 nm thickness.

## Results and discussions

### Pulsed laser deposition

**Figure 1:**
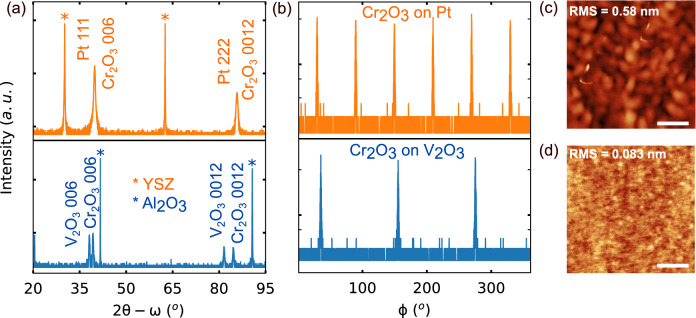
Synthesis of single crystalline Cr_2_O_3_ thin films on V_2_O_3_ electrodes. (**a**) $2\theta -\omega$ X-ray diffraction scans illustrating phase purity and the (0001) orientation of 70 nm thick Cr_2_O_3_ films on (111)-oriented Pt (40 nm)/Ti (4 nm)/YSZ (red) and 30 nm thick Cr_2_O_3_ films on (0001)-oriented V_2_O_3_ (30 nm)/Al_2_O_3_ (blue). (**b**) $\phi$-scans of the {014} peaks of Cr_2_O_3_ reveal the in-plane twinning of the films on Pt/Ti buffered YSZ and single crystallinity on the V_2_O_3_ buffered Al_2_O_3_. (**c**,**d**) AFM topographs of Cr_2_O_3_/Pt/Ti/YSZ and Cr_2_O_3_/V_2_O_3_/Al_2_O_3_. Scale bar: 500 nm. The y-scale for X-ray diffraction data is logarithmic.

Our samples consist of epitaxial Al_2_O_3_ (0001)/V_2_O_3_ (30 nm)/Cr_2_O_3_ (30 to 60 nm) and YSZ (111) (Y_2_O_3_ stabilized ZrO_2_, 8% mole Y_2_O_3_)/Ti (4 nm)/Pt (40 nm)/Cr_2_O_3_ (~ 70 nm) heterostructures. X-ray diffraction (XRD) 2θ-ω scans (Fig. 1a) show phase purity over the scan range and reveal the orientation of the layers. $\phi$-scans of the Cr_2_O_3_{014} planes were then performed to confirm the presence of Cr_2_O_3_ on the Pt layer and the in-plane crystallinity. The $\phi$-scan of the {014} planes will possess three-fold rotational symmetry for single crystal Cr_2_O_3_, thus could not only reveal in-plane film orientation but also identify the single crystal versus twinned nature. As shown in Fig. 1b, the $\phi$-scans present three peaks for Cr_2_O_3_ on V_2_O_3_ and six peaks for Cr_2_O_3_ on Pt, which illustrates a single crystalline Cr_2_O_3_ film and a twinned Cr_2_O_3_ film with 60° in-plane domain rotations, respectively. The surface topography of the films shows a smooth surface for Cr_2_O_3_ grown on V_2_O_3_ (RMS = 0.083 nm) and grainy features for Cr_2_O_3_ grown on Pt (RMS = 0.58 nm) in Fig. 1c,d, respectively. It is important to have a single crystal Cr_2_O_3_ as the crystallographic twin domain boundaries lower the bandgap and results in a reduced breakdown voltage which hinders the electrical switching of the surface magnetization^12,16^. We then evaluate the electrical properties of twinned and single crystal Cr_2_O_3_ films with sub 100 nm thickness to illustrate this improvement.

### Electrical characterizations

**Figure 2:**
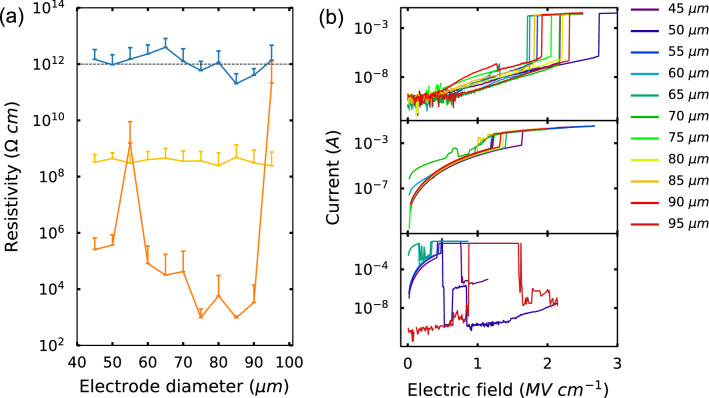
Electrical properties of Cr_2_O_3_ thin films on V_2_O_3_ electrodes. (**a**) Mean resistivity of 60 nm thick Cr_2_O_3_ (blue), 30 nm thick Cr_2_O_3_ (yellow) on (0001)-oriented V_2_O_3_/Al_2_O_3_ and 70 nm thick Cr_2_O_3_ on (111)-oriented Pt/Ti/YSZ (orange). 60 nm thick single crystal Cr_2_O_3_ reaches the bulk resistivity values (dashed black line). Error bars represent the standard deviation. (**b**) Electrical breakdown of low leakage devices in 60 nm thick Cr_2_O_3_ (top panel), 30 nm thick Cr_2_O_3_ on V_2_O_3_/Al_2_O_3_ (middle panel), and 70 nm thick Cr_2_O_3_ on Pt/Ti/YSZ (bottom panel) at different electrode diameters.

Figure 2a plots the mean resistivity of 60 and 30 nm thick Cr_2_O_3_ on V_2_O_3_/Al_2_O_3_ along with 70 nm thick Cr_2_O_3_ on Pt/Ti/YSZ. The mean resistivity at a given electrode size is determined from leakage current measurements of 24 to 25 different capacitors. Error bars represent one standard deviation. Supplementary Figure S1 shows all of the individual data points. The electrode diameters range from 45 to 95 μm. The resistivity of 60 nm thick single crystal Cr_2_O_3_ is ~ 10^12^ $\Omega$ cm and comparable to the bulk value^18^ (dashed black line 10^12^ $\Omega$ cm) and is significantly higher than that of the twinned Cr_2_O_3_. When varying the thickness of single crystal Cr_2_O_3_, the leakage current increases with decreasing thickness. Increasing the electrode area leads to an increase in the number of leaky capacitors which can be accounted for by the increase in probability of encountering a defect. Twinned Cr_2_O_3_ grown on Pt exhibits poor resistivity performance even compared to single crystal Cr_2_O_3_ at lower thickness (30 nm) demonstrating significant improvement of thin film performance when twin domain boundaries are absent.

To further test the dielectric quality of single crystal Cr_2_O_3_ thin films on V_2_O_3_, electrical breakdown tests were performed with capacitors that have high resistivity at different electrode diameters (Fig. 2b). 60 nm thick Cr_2_O_3_ on V_2_O_3_ exhibits a high breakdown field (~ 170 to 225 MV/m—Fig. 2b), which is comparable to previously reported values (200 MV/m) for 500 nm thick Cr_2_O_3_ thin films with 200 × 200 μm electrodes^12^, which is ~ 20% of the bulk breakdown field (1,000 MV/m)^16^. These values, however, are 3 × higher than the breakdown fields observed in the Cr_2_O_3_/Pt heterostructures containing twin domains. Dielectric breakdown of 30 nm thick single crystal Cr_2_O_3_ happens at slightly lower values when compared to thicker films (~ 90 to 160 MV/m) yet remains higher than that observed in the thicker and twinned Cr_2_O_3_/Pt heterostructures.

### Magnetic properties

**Figure 3:**
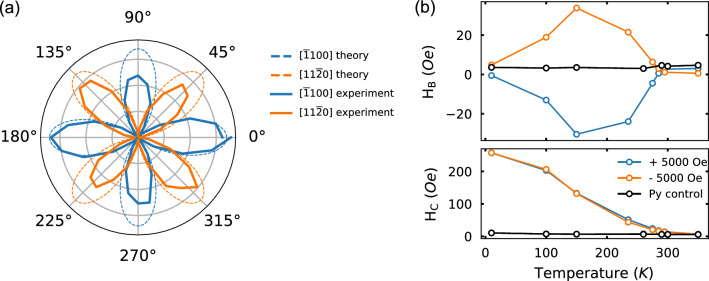
Magnetic characterization of Cr_2_O_3_ thin films on V_2_O_3_ electrodes. (**a**) Optical second harmonic generation intensity from 30 nm thick Cr_2_O_3_ on V_2_O_3_ electrode at room temperature (orange: analyzer along the x-direction ([$11\overline{2}0$]), blue: analyzer along y-direction ([$\overline{1}100$])) and theoretical plot of electric dipole signal (Eq. (1b)) when analyzer is fixed along x-direction (dash-orange) and y-direction (dash-blue) showing the agreement between experimental result and theory. (**b**) Exchange bias field (top panel) and coercive field (bottom panel) as a function of temperature from Pt (5 nm)/Permalloy (Py) (4 nm)/Cr_2_O_3_ (30 nm)/V_2_O_3_/Al_2_O_3_ and Pt (5 nm)/Py (4 nm)/Al_2_O_3_ (labeled Py control) heterostructures. The Py control data shows both $\pm$ 5,000 Oe field cool scans.

We next consider the magnetic properties of the single crystal Cr_2_O_3_ films. To probe the intrinsic magnetic and magnetoelectric order of the 30 nm single crystal Cr_2_O_3_ thin film on V_2_O_3_ at room temperature, we employed transmission optical second harmonic generation (SHG) measurements (Fig. 3a).

Below the Néel temperature, bulk Cr_2_O_3_ possesses the magnetic point group $\overline{3{^{\prime}}}m{^{\prime}}$ that allows the existence of both magnetic (${\chi }_{m}$) and electric (${\chi }_{e}$) dipole susceptibility tensors^19–21^. The magnetic and electric contributions to SHG signals can be expressed as:

1a $$ I_{{MD}}  \propto \left| { - \chi _{m} E_{i}^{2} (\cos 2\;\phi _{i} \sin \;\phi _{s}  + \sin 2\;\phi _{i} \cos \;\phi _{s} )} \right|^{2}  $$

1b $$ I_{{ED}}  \propto \left| { - \chi _{e} E_{i}^{2} (\sin 2\;\phi _{i} \sin \;\phi _{s}  - \cos 2\;\phi _{i} \cos \;\phi _{s} )} \right|^{2}  $$

where *E*_*i*_ is the electric field of the incident beam. When fixing the analyzer along the y-direction ([$$\overline{1}100$$]) (or x- ([$$11\overline{2}0$$])), sin $${\phi }_{s}$$ (or cos $${\phi }_{s}$$) goes to 0, and therefore the SHG intensity signal coming from the magnetic ($${I}_{MD})$$ and electric ($${I}_{ED}$$) dipole contributions will follow sin^2^(2 $$\phi $$) (or cos^2^(2 $$\phi $$)) and cos^2^(2 $$\phi $$) (or sin^2^(2 $$\phi $$)), respectively. The existence of the magnetic dipole signal is attributed to centrosymmetric point group $$\overline{3}$$ m and is expected in single crystal bulk Cr_2_O_3_. The electric dipole signal, however, is a proof of the existence of non-centrosymmetric magnetic order and only exists below the Néel temperature. The rotational-anisotropy data presented in Fig. 3b, following the cos^2^(2 $$\phi $$) (sin^2^(2 $$\phi $$)) dependence on the incident polarization $$\phi $$ when the SHG analyzer is fixed along the y-axis (x-axis), proves the presence of the electric dipole contribution to the SHG signal and confirms that 30 nm thick single crystal Cr_2_O_3_ films on V_2_O_3_ electrodes possess the magnetic symmetry consistent with the magnetic order in bulk Cr_2_O_3_ at room temperature.

Using interface exchange coupling with a thin (4 nm) Permalloy (Py) layer, we probe the blocking temperature of the exchange bias heterostructure to approximate the Néel temperature. Figure 3b shows exchange bias and coercive fields as a function of temperature after cooling from 350 to 10 K in a $\pm$ 5,000 Oe in-plane training field. As the SHG of our thin films is consistent with the c-axis antiferromagnetic anisotropy of bulk Cr_2_O_3_ thin films, the applied in-plane magnetic field while cooling the sample through the Néel temperature is expected to induce a slight canting of the Cr moments in-plane, consistent with the observed in-plane exchange coupling with Py (Supplementary Figure S2). The exchange bias and coercivity enhancement extracted from M(H) curves disappear at ~ 285 K and ~ 295 K, respectively. These data reveal a blocking temperature that is lower than the bulk Néel temperature (307 K), however, this measured blocking temperature is significantly higher than the blocking temperature of films with comparable and greater thickness reported in the literature^22,23^. The blocking temperature can be qualitatively explained using Meiklejohn-Bean model with the competition between interface exchange coupling ($f{J}_{eb}$—where $f$ is a factor between 0 and 1 and represents the degree of interface spin disorder and often assumed to be 1), and the product of magnetic anisotropy energy (${K}_{AF}$) and thickness (${t}_{AF}$) of the antiferromagnetic layer^22,24^. Thus the increase in blocking temperature might be a result of a change in the magnetic anisotropy energy due to the reduced epitaxial strain from the Al_2_O_3_ substrate^22^, however, the measured blocking temperature maybe lower than the Néel temperature of the thin film. Regarding the low temperature behavior, the exchange bias is ~ 0 and begins to increase up to ~ 35 Oe at 150 K where afterward it begins to decrease with increasing temperature up to the blocking temperature. Meanwhile, the coercivity monotonically decreases with increasing temperature. It is reported that there is a change in the Cr_2_O_3_ crystal structure at low temperature that is thought to lead to an in-plane tilting of the magnetic order^9^ or a structural rearrangement at the (0001) surface of Cr_2_O_3_^25,26^ which then affects both its antiferromagnetic structure and surface magnetism. In the Meiklejohn-Bean model, the ratio ($R\equiv \frac{{K}_{AF}{t}_{AF}}{f{J}_{eb}}$) determines the exchange bias and coercive field behavior^24^. The competition between the change in spin structure (affects ${K}_{AF}, {J}_{eb}$) and the surface reconstruction (affects ${J}_{eb}$, $f$) will directly impact the low temperature behavior of the exchange bias field. In order to clarify the situation, future work focusing on isolating these factors is needed.

## Conclusion

In conclusion, by using V_2_O_3_ as an epitaxial buffer layer, crystallographic twinning of Cr_2_O_3_ thin films can be eliminated leading to near bulk dielectric and magnetic behavior in Cr_2_O_3_ films with thickness well below 100 nm. Leakage measurements performed on very thin single crystal Cr_2_O_3_ films, along with electric breakdown tests, as a function of capacitor area suggest the need to further improve film quality or develop additional dielectric layers to mitigate the dielectric parasitics observed at larger capacitor size and enable magnetoelectric characterization with transport methods. Our investigation of magnetic properties in 30 nm thick films indicate bulk magnetic and magnetoelectric order at room temperature and indicate that the magnetoelectric switching of very thin single crystal Cr_2_O_3_ films on V_2_O_3_ electrodes may be possible at room temperature.

## Methods

### Pulsed laser deposition

Samples are fabricated using pulsed laser deposition (PLD) with a 248 nm KrF Excimer laser with a pulse duration of ~ 25 ns using commercially available targets from (V_2_O_5_ 99.9%, Cr_2_O_3_ 99.9%). V_2_O_3_ is deposited onto a Al_2_O_3_ (0001) single crystal substrate at 400 °C from an V_2_O_5_ target with a fluence ~ 2.8 J/cm^2^ and ~ 10 mTorr Ar background pressure. Cr_2_O_3_ is then deposited directly on the V_2_O_3_ film at 500 °C from a Cr_2_O_3_ target with a fluence ~ 2.2 J/cm^2^ under 30 mTorr Ar background pressure. Pt is grown on a (111)-oriented YSZ substrate with a buffer layer of Ti for the purpose of adhesion^27^. Both Pt and Ti are deposited using metallic targets at 550 °C with a fluence ~ 3.6 J/cm^2^ under 20 mTorr Ar gas. Cr_2_O_3_ is then also deposited directly on Pt at 700 °C with a fluence ~ 2.2 J/cm^2^ under 30 mTorr O_2_ background pressure. For testing the magnetic properties of our single crystal thin film, a 4 nm Permalloy (Py) film was then deposited on single crystal Cr_2_O_3_ grown on V_2_O_3_ and was capped with 5 nm of Pt for preventing oxidation. The Py and Pt metal layers are deposited at room temperature with a fluence ~ 3 J/cm^2^ under 20 mTorr Ar gas.

As a Metal–Insulator-Transition material, the growth of V_2_O_3_ has been intensively discussed elsewhere^28–30^. The challenge, however, lies in finding compatible conditions for the growth of Cr_2_O_3_. We found that the presence of oxygen (10 mTorr) as the background gas causes the formation of V_2_O_5_. The V_2_O_5_ was found to melt even at a substrate temperature (650 °C) lower than its melting point (690 °C). Reduction of the substrate temperature in oxygen, however, favors the formation of the VO_2_ phase. By switching to argon gas at the same temperature (400 °C), we achieved V_2_O_3_ thin films with desired topography, x-ray diffraction pattern, and resistivity. The substrate temperature for the growth of the Cr_2_O_3_ layer was chosen to achieve high crystallinity and low surface roughness while mitigating the oxidation of the underlying V_2_O_3_ layer from the oxygen in the ablation plume of the Cr_2_O_3_ target. We optimized our conditions for Cr_2_O_3_ growth on V_2_O_3_ starting from the conditions for high quality Cr_2_O_3_ growth on a bare Al_2_O_3_ substrate. At our deposition energy, Cr_2_O_3_ requires high temperature (700 °C) to achieve good crystallinity. However, at that temperature, we speculate that there is a reaction happening at the interface between V_2_O_3_ and Cr_2_O_3_, since V_2_O_3_ is easy to be oxidized in the presence of oxygen, especially at elevated temperature. The growth temperature was therefore systematically reduced. These above conditions were selected after tuning deposition conditions using surface roughness from AFM and XRD measurements (Peak position, intensity, and oscillation fringes) were used to assess conditions and feedback the growth. From the presence of oscillation fringes around V_2_O_3_ peak, the quality is considered as comparable to previous report of a good crystallinity in thin film^30^.

Circular Ti (6 nm)/Pt (120 nm) top electrode capacitors of diameter from 10 to 150 mμ were defined using a standard liftoff process and PLD deposition.

### X-ray diffraction

2$\theta$ − $\omega$ and $\phi$ scans were performed using a Rigaku Smart Lab diffractometer (Cu *K*_α_ radiation and equipped with a Ge (220) × 2 monochromator on the incident) side to assess the orientation and crystallinity of the films.

### Atomic force microscopy

The surface topography of the films is observed using an NT-MDT NTEGRA atomic force microscope (AFM).

### Magnetometry

Vibrating sample magnetometry was performed using a QuantumDesign Dynacool Physical Property Measurement System (PPMS).

### Electrical measurements

Leakage currents through thin film Cr_2_O_3_ are investigated using a Radiant Technologies Precision Multiferroic II with minimum current detection of 1 pA for a 2 s integration period. An electric field of approximately 1.4 to 1.7 MV/m is applied to detect leakage current. Breakdown tests are performed using a Keithley 2420 with a detection limit of 500 pA. These measurements are performed at ambient conditions in a probe station using W tips with a tip diameter of 5 µm.

### Second harmonic generation measurement

A rotational-anisotropy SHG measurement was performed with the beam at normal incidence. The transmitted SHG intensity is collected with a single photon count detector as a function of the azimuthal angle ϕ_i_ between the incident electric polarization and the in-plane crystalline axis [$11\overline{2}0$] and the analyzer angle ${\upphi }_{\mathrm{s}}$ between the selected SHG electric polarization and the in-plane crystalline axis [$\overline{1}100$]. The incident ultrafast light source was of 800 nm wavelength, 40 fs pulse duration and 200 kHz repetition rate, and focused onto a 20 μm diameter spot on the sample with a fluence of ~ 0.25 mJ/cm^2^.

## Supplementary information

Supplementary Information 1.

## Data Availability

The data that support the findings of this study are available from the corresponding author upon reasonable request.
