# Supplementary material for: Bulk-like dielectric and magnetic properties of sub 100 nm thick single crystal Cr2O3 films on an epitaxial oxide electrode
Source: Sci Rep. 2020 Sep 7;10:14721. doi: 10.1038/s41598-020-71619-1 (PMC7477579; doi:10.1038/s41598-020-71619-1)
Supplement: Supplementary file 1 — Supplementary Information 1. [file 41598_2020_71619_MOESM1_ESM.pdf]

Bulk-like dielectric and magnetic properties of sub 100 nm thick single crystal Cr_2O_3 films on an epitaxial oxide electrode

N. M. Vu[1], X. Luo[2], S. Novakov[2], W. Jin[3], J. Nordlander[4], P. B. Meisenheimer[1], M. Trassin[4], L. Zhao[2], and J. T. Heron[1,*]

[1] Department of Materials Science and Engineering, University of Michigan, Ann Arbor, Michigan 48109, USA

[2] Department of Physics, University of Michigan, Ann Arbor, Michigan 48109, USA

[3] Department of Physics, Auburn University, Auburn, AL 36849, USA

[4] Department of Materials, ETH Zürich, Vladimir-Prelog-Weg 4, 8093 Zürich, Switzerland.

* Author correspondence to jtheron@umich.edu

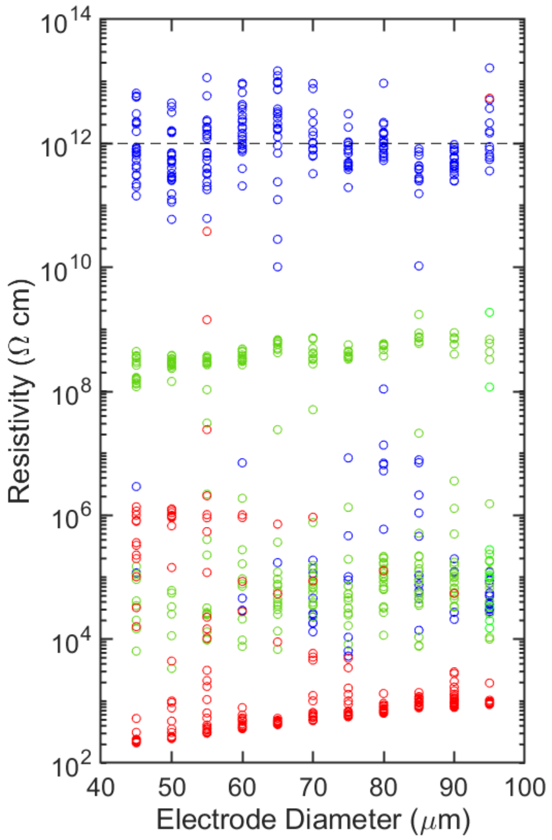

Figure S1. Resistivity of 60 nm thick Cr_2O_3 (blue), 30 nm thick Cr_2O_3 (green) on (0001)-oriented V_2O_3/Al_2O_3 and 70 nm thick Cr_2O_3 on (111)-oriented Pt/Ti/YSZ (red). 60 nm thick single crystal Cr_2O_3 reaches the bulk resistivity values (dashed black line)

‘

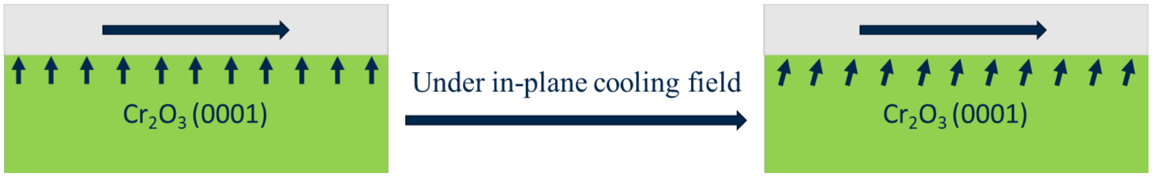

Figure S2. Explanation of in-plane exchange bias between Cr_2O_3 and permalloy
